# Longitudinal progression of cost-related medication non-adherence among Medicare patients with diabetes at high risk of hospitalization: The role of dual eligibility

**DOI:** 10.1371/journal.pone.0329031

**Published:** 2025-08-14

**Authors:** James X. Zhang, David O. Meltzer

**Affiliations:** 1 Department of Medicine, The University of Chicago, Chicago, Illinois, United States of America; 2 Harris School of Public Policy, The University of Chicago, Chicago, Illinois, United States of America; 3 Department of Economics, The University of Chicago, Chicago, Illinois, United States of America; Victoria University, AUSTRALIA

## Abstract

**Objective:**

Little is known about the longitudinal progression of cost-related medication non-adherence (CRN) among the high-need, high-cost diabetes population. We aim to document the longitudinal aspect of CRN among Medicare diabetes patients at high risk of hospitalization and the role of Medicare-Medicaid dual eligibility in CRN.

**Research design and methods:**

617 Medicare diabetes patients at high risk of hospitalization were followed up at 3-month intervals for a total of 16 surveys. Patients’ socio-demographic and health characteristics by dual eligibility were compared using Chi-square tests. The progression of CRN was documented using a Kaplan-Meier Survival Curve. A Cox Survival Regression analysis and a Generalized Estimating Equation (GEE) analysis were conducted to evaluate the adjusted hazard ratio (HR) and population-averaged effect of dual eligibility on CRN, controlling for socio-demographic and health characteristics.

**Results:**

303 patients (49.1%) reported dual eligibility, among whom 151 (49.8%) reported CRN; they were more likely to be under 65 (p < 0.01), had lower income (p < 0.01), were less likely to report cardiovascular disease (p = 0.05), and were less likely to report CRN (p < 0.01) compared to those who did not report dual eligibility. Those with dual eligibility had a lower hazard ratio (HR = 0.67, p < 0.01) and lower likelihood of reporting CRN (coefficient = −0.40, p < 0.01), and those with depression had higher hazard ratio (HR = 1.31, p = 0.03) and higher likelihood of reporting CRN (coefficient = 0.32, p < 0.01) in the Cox model and GEE, respectively.

**Conclusions:**

While insurance coverage enables patients to overcome their major deficiency in income, many patients fall through the cracks as their disease progresses. Depression is a major risk factor for CRN. Health policy addressing CRN needs to be implemented in tandem with clinical intervention, targeting those at the increasing risk of CRN.

## Introduction

The cost barrier to medications has been a serious challenge for millions of Americans [1]. One in four adults in the US has a difficult time affording their medications [2], and among older adults with diabetes, 19% cut back on medication use in the prior year due to cost (as reported in 2004) [3]. Although the Medicare Part D outpatient prescription drug program, which went into effect on January 1, 2006, aimed to improve the access to and affordability of prescription drugs for the elderly and adults with disabilities, research using cross-sectional data sets over time found that cost-related medication non-adherence (CRN) has been persistent or even increased among the sickest Medicare beneficiaries, including the elderly with complex medical needs and people with disabilities [4–7]. While these cross-sectional estimates showed the aggregate trend in CRN for the populations studied, because the subjects were not longitudinally linked up, the progression of CRN over time by the same subjects is unclear.

Diabetes is a progressive condition with worsening complications over time, hence a single cross-sectional study fails to capture the gravity of CRN as patients struggle increasingly with worsening health, increasing need for medication, and deteriorating economic wellbeing at the same time. This calls for longitudinal study of the progression of CRN among diabetes patients at risk of CRN, which has not been seen in the literature.

Diabetes patients with Medicare-Medicaid dual eligibility may be particularly at risk of CRN because many of them have multiple conditions and disabilities and are simultaneously economically disadvantaged. Nationwide, twelve million Americans are dually eligible for Medicare and Medicaid benefits; they represent 20% of all Medicare beneficiaries and 15% of all Medicaid beneficiaries but account for one-third of total expenditures for each program [8]. The dual coverage of Medicare and Medicaid helps pay the premium and coinsurance for prescription drugs, which reduces out-of-pocket payments [9]. Dual-eligible patients are at the lowest level of the economic ladder and can be extremely sensitive to out-of-pocket payments, and many dual-eligible patients are in managed-care prescription drug programs with higher out-of-pocket payments for drugs outside their formularies, in the Medicare program in general and in the state of Illinois through its Medicare-Medicaid Alignment Initiative (MMAI) specifically [10,11]. Thus how these dual-eligible patients fare in terms of CRN over time compared to their non-dual-eligible Medicare counterparts is an important practice and policy question.

The progression of CRN and the role of dual eligibility is rarely examined with direct comparison in a defined high-cost patient population such as Medicare population, although research reported high prevalence rates of CRN among dual eligibles in exploratory cross-sectional studies and short-term longitudinal follow-up [12,13]. The role insurance coverage plays particularly in terms of offsetting the access barrier due to low income among diabetes patients is thus largely unknown. Identifying and mitigating the high risk of CRN prevents patients from falling into a vicious cycle of non-adherence, poorer health, more intensive and expensive care, and thus even worse non-adherence. This cycle results in even greater costs to society and foregone treatment opportunities for patients. Therefore, understanding the role of dual eligibility in CRN is significant to all stakeholders including patients, practitioners and policy makers.

We therefore proposed to study the longitudinal aspect of, and the role of dual eligibility on, CRN over time in a sample of Medicare beneficiaries with high risk of hospitalization. This group of patients has been known to have high resource utilization, and hence it is even more pressing to understand the progression in CRN over time as non-adherence to medication may result in much worse health and higher downstream costs. We also aimed to identify the potential risk factors for CRN longitudinally by analyzing patients’ sociodemographic strata and health characteristics as we are interested in the offsetting effect between dual eligibility and other individual characteristics.

## Research design and method

We used a sample of 617 Medicare diabetes patients at high risk of hospitalization who had previously enrolled in a study of the Comprehensive Care Physician Model (CCP) conducted in an urban academic medical center [14]. The original cohort of 2000 subjects were enrolled based on the criteria of having been hospitalized at least once in the past year or being in the emergency room for care at the time of enrollment and being a Medicare beneficiary. The purpose of the CCP study is to evaluate a care model that integrates physician services at the ambulatory and inpatient stages so that the care provided to patients is “comprehensive”. To qualify for such a care model economically, the patients needed to be at high risk of hospitalization and hence high resource utilizers. Our internal analysis indicated that based on the enrollment criterion these patients were at 300−400% of the average annual health cost for Medicare beneficiaries during the study’s follow-up period [10]. CRN rates among this group of patients are likely higher than the general population because the study’s inclusion criteria mean that the patients likely have higher resource utilization and severity of illness [15]. Working with this population allows us to study the progression of CRN for those at heightened risk. For this study, only those who self-reported as having diabetes at enrollment were included. The study period is between 2012 and 2019, when the national economy was steadily recovering from the Great Recession in 2008 and before the COVID-19 pandemic. This provided a clear picture of patients’ CRN behaviors without major economic downturn or pandemic, which may alter the CRN progression. Although the CCP study itself is a randomized controlled trial, for this study we pooled the data of the study arm and control arm, as our interest is primarily in tracking the pattern of CRN rather than in the effect of the CCP on CRN over time. We nevertheless controlled for the study arm in the multiple regression analysis to reflect the population-average CRN adjusted for other confounding factors.

The study period was between November 1, 2012, and December 30, 2019. Our study included up to 16 surveys of CRN for each subject in 3-month follow-up intervals through the end of 2019; fewer if subjects were deceased or dropped out during the follow-up period. There was a total of 680 subjects who self-reported diabetes during the recruitment process between March 15, 2013, and June 23, 2016. We excluded 3 subjects who eventually withdrew from the study, and another 28 subjects who were deceased during the first 3 months after enrollment. For the 649 subjects who survived the first quarter after enrollment, 32 subjects did not complete any follow-up surveys. The overall response rate for the follow-up surveys was hence 95%, and the final study sample size was 617. To fully capture the CRN progression for the complete study sample, we kept 164 subjects who died during the follow-up period but had at least one follow-up survey.

CRN was self-reported based on four questions adapted from the survey questionnaires of the Medicare Current Beneficiary Survey, with a recall timeframe set at 3 months instead of 1 year. Patients were asked whether, during the past three months, they had ever done the following due to cost: 1) not fill or refill a prescription, 2) delay filling a prescription, 3) skip doses, or 4) take smaller doses to make medication last longer [16]. CRN then was categorized as 1 if the patients reported any of these 4, and 0 if none. Patients who did not answer CRN questionnaires at any point during the 48-month follow-up period were treated as not reporting CRN for that observation. Among the total of 6,628 surveys completed by those 617 patients, 237 (3.6%) indicated “don’t know” or “refusal” to one or more of the four CRN questions for the survey. Theoretically, the 3-month timeframe may reduce recall bias and provide more detailed information about CRN behavior than 12-month recall questionnaires. Over time, there are 16 time points of CRN from the first survey to the end of the 48-month follow-up.

To evaluate the role of dual eligibility on prevalence of CRN, we first developed a survival analysis to analyze by dual eligibility the increasing rate of subjects reporting CRN over time, taking account of right-censoring due to death and dropouts. Kaplan-Meier survival curves were drawn, and p-value was obtained for the hazard ratios for the comparison of the two groups with and without dual eligibility. This analysis aimed to document the distinct progression of CRN by patients’ baseline dual eligibility, considering right-censoring of data due to death and dropouts. In this analysis, the outcome variable has both an event (i.e., CRN) and a time value associated with it (i.e., time to reporting CRN), while addressing the fact that patients might be right-censored due to death or dropout and thus have no observed true survival. Hence, overall, the cumulative probability of reporting CRN increases over time for each group. This analysis allows us to ascertain the gross effect of dual eligibility in terms of preventing patients from falling into CRN after they were enrolled in the study. We further conducted a Cox survival regression analysis evaluating the adjusted hazard ratio of dual eligibility by controlling for other potential confounders including socio-demographic and health characteristics collected at baseline in the survey when patients were enrolled. These factors included: age, sex, race, education, health literacy [17], educational attainment, income, self-reported health, health conditions (cancer, cardiovascular disease including angina, congestive heart failure and coronary artery disease, depression, and kidney disease), questionnaires on limitations in functional status including Activities of Daily Living (ADL) and Instrumental Activities of Daily Living (IADL) [18,19], and study group (standard of care vs intervention). These factors have been known to be risk factors for CRN in cross-sectional studies [16,20,21], but how they impact CRN longitudinally has only been recently explored among the high-cost, high-resource-utilization Medicare population [15]. We cross-checked the self-reported birthdates in the survey with the clinical information system and linked Medicare enrollment files to ensure the calculated age variable reflect the best information. The race variable was self-reported, considered the gold standard in research [22]. It is important to note that dual eligibility was reported at enrollment. Regarding income, the questionnaire asked, “Please point to the income category that best describes (your/PATIENT’S) total household income before taxes (include salary, social security, pension, and other income)?” The income categories go from $2,000 or less, $2,501 to $5,000, $5,001 to $25,000 in increments of $5,000, then $25,000 to $35,000, $35,001 to $50,000, $50,001 to $100,000, $100,001 to $200,000, and over $200,000. “Don’t know” or “refusal” to the question were also noted. By controlling for those factors, the role of dual eligibility in preventing patients from falling into CRN can be further ascertained by reducing confounding, and the offsetting effect of income and insurance coverage can be determined. Because many patients did not report income, a common phenomenon in social science/health services research [23], we created a separate variable indicating “don’t know or refusal” in the regression analysis to separate those patients from those who had zero income. We also collapsed the more detailed income categories in the survey to <$15k, $15k ~ $25k, > $25k to reduce the random errors in reporting those incomes. The < $15k is closely associated with the Federal Poverty Guideline [24]. Similarly, for educational attainment, although the number of “don’t know or refusal” is much smaller, we created another indicator variable to separate those respondents from others in the regression analyses. Since health literacy may play an important role in health-seeking behavior with respect to understanding the efficacy and side effects of the medication (15), we have included this variable in the regression analysis.

Since our data included repeated measurement of CRN on a quarterly basis for up to 16 quarterly follow-ups, we developed a generalized estimating equation model (GEE) to assess the population-averaged effect of the risk factors on CRN, considering correlations among repeated observations of the patients, which is quite often unknown [25]. One strength of such an approach is lower variability and thus more efficient comparison, allowing us to detect a difference within socio-economic strata in a sample with modest size. The GEE model uses a binomial family function, a probit link function, and an exchangeable correlation structure to address the binary outcome variable and correlation among the longitudinal follow-ups of the patients. There is no order effect in the repeated measures in this analysis, as patients can report CRN intermittently, and the research has shown patients are not always persistent in CRN [15]. Our examination of the average effect of each risk factor on CRN gives us further evidence about the relationship between dual eligibility and CRN while holding other variables constant, and about the offsetting effects among those variables. The Cox regression and GEE analysis are two complementary analyses as the former highlights the role of dual eligibility in preventing patients from falling into CRN, which is a time-to-event measure, and the GEE illustrates that while multiple CRN could be reported over time, which can be intermittent, transient, and persistent depending on the relative frequency of CRN [15], the population-averaged effect of dual eligibility were averaged taking into account all occurrences of reported CRN and the correlation among the repeated observations in contrast to the Cox model which shows the time to the first occurrence of the first reported CRN after enrollment. In this sense, the Cox model shows the preventive aspect of dual eligibility, and the GEE shows the maintaining aspect of dual eligibility. Both models have the same set of covariates. The analysis was conducted using STATA 15.0 (StataCorp LLC, College Station, TX, USA).

The study was approved by the University of Chicago Institutional Review Board #12–1440. The consent was informed and written for the recruitment of the original study subjects of 2,000 patients in the Comprehensive Care Physician Study. For this study which involved secondary-data analyses of 617 subjects, the consent was waived.

## Results

For those 617 subjects, a total of 6,628 follow-up surveys were completed, with median of 12 and mean of 10.7 (s.d. 5.2) surveys completed per person. 186 (41%) of the 453 subjects who were alive at the end of 4-year follow-up completed all 16 follow-up surveys. Table 1 shows the patient demographics and health characteristics stratified by dual eligibility for the 617 subjects. 303 patients (49.1%) reported dual eligibility, and compared to those who did not report dual eligibility, those with dual eligibility were more likely to be under the age of 65 (p < 0.01), had lower income (p < 0.01), and were less likely to report having cardiovascular disease (p = 0.05). Overall, 193 non-dual-eligible patients (61.5%) and 151 dual-eligible patients (49.8%) reported CRN at least once during the 16-quarter follow-up period.

**Table 1:** Patient Socio-Demographic and Heath Characteristics.

	*Dual Eligibility*		
	*Dual eligible n (%)*	*Non-dual-eligible* *n (%)*	*P-value*
Total Patients	303	314	
Age			
< 65 years	158 (52.2)	106 (33.8)	<0.01
≥ 65 years	145 (47.9)	208 (66.2)	
Sex			
Male	101 (33.3)	120 (38.2)	0.19
Female	202 (66.7)	194 (61.8)	
Race			
White	7 (2.3)	18 (5.7)	0.07
Black or African American	278 (91.8)	277 (88.2)	
Other race	18 (5.9)	19 (6.1)	
Ethnicity			
Hispanic or Latino	8 (2.6)	15 (4.8)	0.16
Non-Hispanic	295 (97.4)	299 (92.2)	
Education			
< High School	91 (29.7)	86 (27.4)	0.44
High School	90 (29.6)	82 (26.1)	
Some College	84 (27.6)	91 (29.0)	
College Graduate	31 (10.2)	46 (14.7)	
Don’t know/refused	7 (2.3)	9 (2.9)	
Limited Health Literacy			
Extremely or quite confident filling out medical forms	126 (41.6)	133 (42.4)	0.90
Somewhat/a little bit/not at all filling out medical forms	173 (57.1)	178 (56.7)	
Don’t know/refused	4 (1.3)	3 (1.0)	
Income			
< $15k	153 (50.5)	104 (33.1)	<0.01
$15-$25k	30 (9.9)	39 (12.4)	
≥ $25k	12 (4.0)	60(19.1)	
Don’t know/refused	108 (35.6)	111 (35.4)	
Self-Reported Health			
Good, Very Good or Excellent	100 (33.0)	106 (33.8)	0.84
Fair or Poor	203 (67.0)	208 (66.2)	
Health Conditions			
Cancer	26 (8.6)	25 (8.0)	0.78
Cardiovascular Disease	134 (44.2)	164 (52.5)	0.05
Depression	94 (31.0)	81 (25.8)	0.15
Kidney Disease	89 (29.4)	95 (30.1)	0.81
Limitations in Functional Status			
Activities of Daily Living: Mean (SD)	1.4 (1.8)	1.2 (1.7)	0.16
Instrumental Activities of Daily Livig: Mean (SD)	3.2 (2.7)	3.2 (2.8)	0.94
Study Group			
Intervention	145 (47.9)	158 (50.3)	0.54
Standard of Care	158 (52.2)	156 (49.7)	
Reporting CRN At Least Once	151 (49.8)	193 (61.5)	<0.01

Legend: p-value derived from Chi-squared tests, respectively, except for limitations in functional status, where the p-values were derived by two-tailed t-tests.

**Fig 1:**
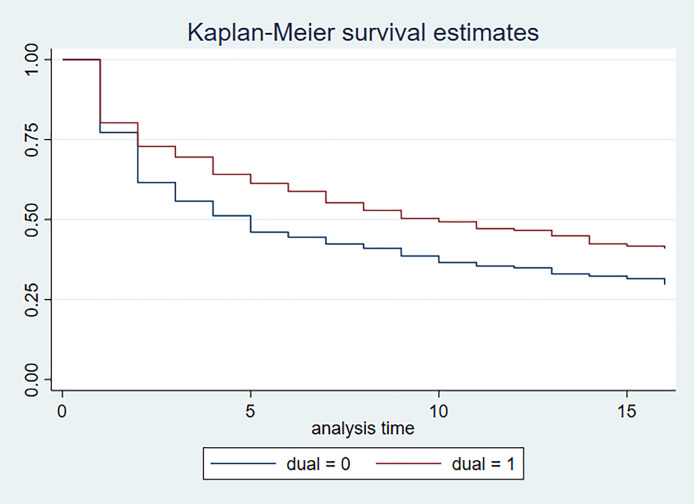
Kaplan-Meier survival estimates for reporting cost-related medication non-adherence by dual eligibility in 16 quarters. Legend: p < 0.01.

Fig 1 shows the Kaplan-Meier survival estimates for reporting CRN by dual eligibility in 16 quarters. Those with dual eligibility consistently had lower cumulative probability of reporting CRN over time (p < 0.01), although more than half patients in the dual eligible and non-dual-eligible groups eventually reported CRN, which increased over time. Non-dual-eligible patients reached that threshold with 5 quarters and dual-eligible patients with 10 quarters, considering right-censoring due to deaths and dropouts.

Table 2 shows the hazard ratios (HR) from the Cox survival regression and coefficients from the GEE analysis and their 95% confidence intervals and associated p-values for dual eligibility and other risk factors, respectively. Dual eligibility is associated with lower hazard ratio (HR = 0.67, p < 0.01) and lower likelihood of reporting CRN (coefficient = −0.40, p < 0.01) in Cox regression and GEE, respectively. In addition, depression is associated with higher hazard ratio (HR = 1.31, p = 0.03) and higher likelihood of reporting CRN (coefficient = 0.32, p < 0.01) in the Cox model and GEE, respectively. Compared to those with household income > $25k, those with less than <$15k and between $15k and $25k had a tendency of higher hazard ratio in the Cox model (1.21 and 1.41, respectively), with p-value of 0.34 and 0.15, respectively, and a similar pattern in the GEE model (positive coefficients of 0.16 and 0.35, respectively), with p-value of 0.38 and 0.13, respectively. Good, very good, and excellent self-reported general health had a tendency of lower hazard ratio of 0.81 (p = 0.09), and a negative coefficient of −0.18 (p = 0.13) in the Cox regression and GEE analysis, respectively.

**Table 2:** Associations between socio-demographics and health characteristics to CRN outcomes.

	*Cox Survival Regression*		*Generalized Estimating Equation*	
	*HR (CI)*	*P-value*	*Coef. (CI)*	*P-value*
Dual Eligibility (Ref: Medicare only)	0.67 (0.53, 0.84)	<0.01	−0.40 (−0.62, −0.19)	<0.01
Age (Ref: ≥ 65 years)				
< 65 years	1.22 (0.97, 1.53)	0.10	0.15 (−0.08, 0.37)	0.19
Sex (Ref: Female)				
Male	0.90 (0.71, 1.14)	0.38	−0.15 (−0.37, 0.07)	0.19
Race (Ref: Black)				
White	0.79 (0.41, 1.51)	0.46	−0.28 (−0.82,0.26)	0.30
Other race	1.21 (0.74, 1.97)	0.46	0.21 (−0.27, 0.69)	0.39
Hispanic or Latino/a	0.74 (0.38, 1.47)	0.40	−0.27 (−0.88, 0.34)	0.39
Education (Ref: Some College)				
< High School	0.92 (0.68, 1.24)	0.60	−0.08 (−0.36, 0.21)	0.60
High School	1.06 (0.80, 1.41)	0.68	0.05 (−0.22, 0.33)	0.71
College Graduate	0.99 (0.69, 1.43)	0.95	0.06 (−0.30, 0.41)	0.76
Limited Health Literacy	1.05 (0.84, 1.31)	0.65	0.12 (−0.11, 0.35)	0.32
Income (Ref: ≥ $25k)				
< $15k	1.21 (0.82, 1.80)	0.34	0.16 (−0.20, 0.53)	0.38
$15-$25k	1.41 (0.89, 2.23)	0.15	0.35 (−0.10,.81)	0.13
Self-Reported Health (Ref: Fair or Poor)				
Good, Very Good or Excellent	0.81 (0.63, 1.03)	0.09	−0.18 (−0.41, 0.05)	0.13
Health Conditions				
Cancer	1.08 (0.73, 1.60)	0.69	0.10 (−0.27, 0.48)	0.59
Cardiovascular Disease	0.87 (0.70, 1.09)	0.22	−0.20 (−0.41, 0.00)	0.06
Depression	1.31 (1.03, 1.67)	0.03	0.32 (0.08, 0.56)	<0.01
Kidney Disease	0.96 (0.76, 1.23)	0.77	−0.10 (−0.32, 0.13)	0.19
Limitation in Functional Status				
Activities of Daily Living	0.95 (0.87, 1.03)	0.24	−0.05 (−0.13, 0.03)	0.19
Instrumental Activities of Daily Living	1.00 (0.95, 1.05)	0.98	−0.02 (−0.07, 0.03)	0.51
Study Group (Ref: Standard of Care)				
Intervention	0.90 (0.72, 1.12)	0.33	−0.11 (−0.31, 0.10)	0.31

## Discussion and conclusions

This is the first study that follows diabetes patients longitudinally on a quarterly basis to track the progression of reporting CRN and assess the population-averaged effect of risk factors on reporting CRN using repeated measurements. We found the prevalence rates of reporting CRN in both dual-eligible and non-dual-eligible Medicare diabetes patients at high risk of hospitalizations were triple the CRN prevalence rate among all U.S. adults with diabetes (estimated at 16.5% for 2013–2014 using cross-sectional analysis) [26]. This raises questions about the adequacy of cross-sectional analysis to fully capture the progression and gravity of CRN among diabetes patients as their disease conditions progress and about how policy and practice can be improved to mitigate CRN.

It is encouraging to find that dual-eligible patients had a relatively lower CRN prevalence rate at each time point when compared to those non-dual eligible, despite dual-eligible patients having much lower income. It should be noted that a substantially larger fraction of dual-eligible diabetes patients had household income under $15k, and a substantially smaller fraction had household income higher than $25k compared to their non-dual-eligible counterparts. Nevertheless, the dual-eligible diabetes patients were less likely to report CRN in both time-to-event and population-averaged likelihood. This shows that insurance coverage enables patients to overcome their major deficiency in income, which is consistent with the Anderson Health Utilization model (though the Anderson model does not specifically or explicitly address the offsetting effects of different factors) [27]. However, while Medicare-Medicaid dual eligibility is protective for preventing diabetes patients from falling into CRN, the survival analysis demonstrated that more than half of them report CRN as their diseases progress. This suggests that many patients fell through the cracks and current insurance coverage alone may not be sufficient to help patients overcome cost barriers to medications over time.

In this study, we found that depression is a major risk factor for CRN. Although this is not the primary purpose of the study, we note it here because literature suggests that this can be an important factor in treating diabetes and reducing CRN [28]. When a patient is depressed, the outlook for their disease management may appear dim, adherence to medication is less motivated, and the cost barriers may be magnified. A cross-sectional study found that depressive symptoms increased the odds that patients cut back on medications for diabetes care [28]. This study advanced the understanding of the effect of depression on CRN as the complementary evidence of such a risk factor in survival analysis and GEE in this study suggested that depression may have a temporal effect on patients’ CRN behaviors; that is, it propels them to be engaged in CRN sooner, and it has a lasting effect on their reporting CRN over time. This also suggests that CRN behaviors may be affected by mental health broadly, as the value of medication treatment is based on patients’ internal valuation, judgment, and decision-making process. In addition, it is possible that their depressive state makes it more likely to report CRN even if the non-adherence was not actually cost-related. This highlights the importance for medical practice to address depression among diabetes patients to improve their wellbeing and reduce CRN. This finding provides an important insight into addressing this policy issue in tandem with clinical intervention.

It should be noted that while dual-eligible patients receive generous insurance coverage for premium, copayments, and co-insurance for their medications, due to the implementation of managed care programs among state Medicare-Medicaid alignment initiatives, many dual-eligible patients may be subject to large out-of-pocket payments for medication if the drugs are not in the programs’ formularies [15]. Hence, it is also possible that dual-eligible patients reporting CRN may reflect sensitivity to those payments. More research is needed to investigate the utilization management tools in managed care programs among the dual-eligible, and to evaluate the interaction among income, insurance, and price sensitivity in affecting CRN among diabetes patients in general.

This study demonstrated that providing insurance coverage alone may not be sufficient to reduce CRN as the risk for CRN is multi-faceted, for example, depression is found to be a significant risk factor, and depression may interact with the patients’ perception of economic burden and exacerbate CRN. This finding provides an important insight into addressing this policy issue in tandem with clinical intervention.

By addressing right-censoring due to death and dropouts in its survival analysis, this study found that more than half of dual-eligible and non-dual-eligible patients in this high-risk population would have reported CRN much sooner and ended higher than the prevalence rate of those who were alive and reporting. This suggests that death may be a competing risk for reporting CRN, and had patients lived longer, even more may eventually have reported CRN. Research has shown that Medicare patients have high resource utilization during their last year of life [29,30], and many diabetes patients suffer from premature death due to heart disease and other complications. More research is needed to understand the struggles of diabetes patients against diseases and cost barriers to pay for needed care when they approach the end of life.

Our study benefited from the fact that dual-eligible patients were over-represented in the sample compared to the national average, because the patients serviced by our medical center are predominantly low-income minorities. Rarely does a nationally representative sample have such a large size of dual-eligible diabetes patients at high risk of hospitalization who have been persistently followed up for longitudinal study. Our study shows that when diabetes patients are followed up longitudinally, their progression in CRN accumulates irrespective of their dual eligibility. This demonstrates the importance of examining CRN longitudinally as it shows that diabetes patients are increasingly strained by economic resources as they need to pay more and perhaps use more expensive drugs. Policy and clinical intervention should target those at the increasing risk of CRN especially in the light of post-pandemic inflation and significant policy changes that reduced unemployment and welfare benefits for the poor that have occurred since the study period.

This study has several limitations. First, self-reporting is always affected by recall bias and may also be subject to a sense of shame when reporting CRN, and hence we could be underestimating CRN rates [15]. Such recall bias was reduced in this study as we set the recall timeframe at 3 months instead of the 12-month timeframe found in most national estimates in the literature. The short 3-month recall period for follow-surveys may reduce the “forgetting” of CRN seen in many annual national CRN surveys, as research suggests that people tend to “forget” the events in the first half of year in retrospective data [31]. Future research should aim at developing objective measurement of CRN such as claim-based to complement the subjective reporting of CRN [32]. Second, this study included patients cared for at an urban academic medical center with a majority African American patient population, thus it may not be generalized to the entire Medicare population. While it provides insight into an important patient population with dual eligibility of which the national datasets often lack sample size in the context of CRN, caution needs to be exercised in interpretation due to geographic variation in CRN rates given different Medicare-Medicaid dual eligibilities across states. Third, while this study examined the time to reporting CRN and the population-averaged effect of dual eligibility and other risk factors, it does not address the persistence of CRN, i.e., the frequency of reporting or intensity of CRN. The persistence of CRN is a complex phenomenon, warranting dedicated study. Patients may have transient and/or intermittent CRN in addition to persistent CRN [15], and the sample size of this study will not allow us to detail a complex pattern with these many covariates. Fourth, the study does not track the seasonal variability of CRN as it requires another time parameter. Our internal conversation with patients suggested that giving gifts during the holiday season depletes elderly patients’ cash reserves and raises financial management concerns. Future care models may need to incorporate social services in response to such concerns [15]. Fifth, the study period is between 2012 and 2019, when the national economy was steadily recovering from the Great Recession in 2008 and before the COVID-19 pandemic. Our recruitment process of this sample was through March 15, 2013, and June 23, 2016; hence the expansion of the Medicaid coverage through Obamacare starting at January 1, 2014, in the state of Illinois was captured to a large degree at the baseline. Research has shown impact of Obama Care on CRN [33], and its external impact on CRN is beyond the scope of this study. Sixth, we have limited power to detect differences by socio-economic strata despite a relatively large sample size, as the estimations for factors such as income and general health had the expected directional sign but did not reach statistical significance in the Cox model and GEE analysis, respectively. It is also possible that the income effect is non-linear, given the offsetting effect of varieties of insurance coverage (particularly public insurance coverage for the poor). Future research should be directed at examining nuanced offsetting effects between income and insurance coverage over time. Seventh, patients who didn’t answer CRN questionnaires were treated as “not reporting CRN,” which could underestimate true CRN rates. About 3.6% of surveys had “don’t know” or “refusal” responses to CRN questions. Although the direction of bias is less certain given multiple reasons for not reporting CRN such as shame or lack of trust in reporting the matter, future research should aim to explore the direction and magnitude of bias when a larger sample is available. Finally, while we captured the impact of death on CRN through a new approach to evaluating the longitudinal pattern of CRN by survival analysis, our sample size limited our ability for a separate analysis for those alive and deceased in the GEE estimation. Medicare patients have high resource utilization during their last year of life [29,30,34], and future research should aim at advancing understanding of patients’ economic struggles at end of life.

In summary, while insurance coverage enables patients to overcome their major deficiency in income, many patients fall through the crack as their disease progresses. Depression is a significant risk factor, and depression may interact with the patients’ perception of economic burden and exacerbate CRN. Health policy addressing CRN needs to be implemented in tandem with clinical intervention, targeting those at the increasing risk of CRN.

## Supporting information

S1 Data Analytic data for sample characteristics, survival analysis, and GEE estimation.
(DTA)

S2 Data Analytic data for sample characteristics, survival analysis, and GEE estimation.
(DTA)

S3 Checklist (PDF)
